# Multilevel view on chromatin architecture alterations in cancer

**DOI:** 10.3389/fgene.2022.1059617

**Published:** 2022-11-18

**Authors:** Maria Gridina, Veniamin Fishman

**Affiliations:** The Federal Research Center Institute of Cytology and Genetics, Siberian Branch of the Russian Academy of Sciences, Novosibirsk, Russia

**Keywords:** cancer, 3D genome organization, chromatin organization, chromosome territories, topologically associated domain

## Abstract

Chromosomes inside the nucleus are not located in the form of linear molecules. Instead, there is a complex multilevel genome folding that includes nucleosomes packaging, formation of chromatin loops, domains, compartments, and finally, chromosomal territories. Proper spatial organization play an essential role for the correct functioning of the genome, and is therefore dynamically changed during development or disease. Here we discuss how the organization of the cancer cell genome differs from the healthy genome at various levels. A better understanding of how malignization affects genome organization and long-range gene regulation will help to reveal the molecular mechanisms underlying cancer development and evolution.

## Introduction

The genome is distinctly organized in the nucleus by various architectural proteins. This organization is essential for genome functioning by the colocalization of genes and the regulatory elements, as well as by the establishment of efficient replication origins and an initiation repair-prone chromatin compartment. Cancer driver mutations, including structural variations and point mutations, lead to a global disruption of the cell program that has an impact in destruction of the genome organization.

Understanding how chromosomes are folded in the nucleus is a key issue in molecular biology. Chromosomes occupy separate but interacting chromosome territories within the cell nucleus. Euchromatin and heterochromatin are two major types of chromatin into which all chromosome territories can be divided. According to genome-wide chromatin conformation capture (Hi-C) data, they correspond to compartments A and B, respectively (Lieberman-Aiden et al., 2009). Compartment segregation correlates with gene activity, chromatin accessibility, histone marks, replication time and reflects spatial separation of different chromatin types, since compartments interact with distal regions of the same type. These long-range interactions reflect the assembly of functional units within the nucleus, including transcription or replication factories and splicing speckles. At the sub-megabase scale, high frequency contact areas form topologically associating domains (TADs). The interactions between cis-regulatory elements and their target promoters are facilitated within these domains. At the same time, such contacts of promoters with non-specific regulatory elements outside TADs are prohibited due to the insulatory function of TAD boundaries. As a result, TADs allow fine-tuning of expression programs during development and differentiation.

Here we describe evidences of chromatin architecture alterations at different levels in cancer. Studies of chromosomal territories, chromatin compartments and topologically associating domains reveal key elements of transformed genome structure in cancer. Our analysis highlights the relationship between the structure and function of the genome in human cancer.

### Level 1—Nucleus

The abnormal morphology of the nuclei is one of the attributes that is widely used to identify malignant cells. The most easily assessed feature is nuclear size, which is usually increased in malignant cells (Heras et al., 2013). The mechanisms underlying changes in nuclei size are not fully understood. The simplest explanation assumes that nuclear size changes due to ploidy alterations, often observed in cancer cells (Weaver & Cleveland, 2006). However, this is not a satisfactory explanation, and it should be considered only as a correlation and not a causal relationship, as different somatic cell types having the same ploidy can have different nuclear sizes. In cancer cells, the correlation between of ploidy and nucleus size is relevant only for some cancer types, for example: prostate, breast, lung, colonic adenocarcinoma (Jevtić and Levy, 2014). Moreover, it is enough to overexpress specific oncogenes to induce increasing nuclear size without DNA ploidy changes: p300 in prostate cancer cells (Debes et al., 2005), RET tyrosine kinase in thyroid carcinoma (Fischer et al., 1998). Changes in the nucleus volume can lead to alterations of the nuclear proteins, DNA and RNA concentration. As a result, this can lead to dramatic variations in the activities of RNA and DNA polymerases, transcription factors, the ability of different chromosomal domains to communicate with each other, the assembly of key structures, and priming of cancer development (Webster et al., 2009).

In addition to the changes in size, cancer cell nuclei often display an atypical morphology, which supposes defects in the nuclear envelope structure. The inner layer of the nuclear envelope is the nuclear lamina, which is formed by a network of filament proteins called A- and B-type lamins. The proper expression of lamin genes is important to maintain the correct rigidity of the nucleus. Downregulation of lamin genes makes nuclei softer and increases nuclear deformability leading to the appearance of invaginations and nuclear blebbing (Lammerding et al., 2004; Shimi et al., 2008; Guilluy et al., 2014), whereas their overexpression increases nuclear rigidity (Ferrera et al., 2014; Lavenus et al., 2022). Dysregulation of lamins is often observed in a variety of human tumors. For example, B-type lamin overexpression was shown for melanoma, pancreatic and prostate cancer (Li et al., 2013; Luo et al., 2021; Lämmerhirt et al., 2022). A-type lamins are upregulated in ovarian cancers (Wang et al., 2019). Whereas their reduced expression is typical for small cell lung carcinoma, leukemias, lymphomas, breast cancer, colon cancer, gastric carcinoma and ovarian carcinoma (Broers et al., 1993; Agrelo et al., 2005; Wu et al., 2009; Wazir et al., 2013; Alhudiri et al., 2019; Jia et al., 2019). Thus, the observed changes in the morphology of the nuclei of cancer cells may be the result of lamin dysregulation.

On the other hand, atypical morphology may be caused by changes in chromatin organization. In most of the eukaryotes, the heterochromatin is located on the periphery of the cell nucleus (Falk et al., 2019). It is believed that peripheral heterochromatin location is important for gene silencing (Akhtar and Gasser, 2007; Steensel and Belmont, 2017). At the same time, such location of heterochromatin can increase the structural stability of the nucleus and its ability to resist mechanical forces, for example, during cell migration (Gerlitz & Bustin, 2011). Increasing euchromatin by treatment of mammalian cells with histone deacetylase inhibitors (valproic acid, or trichostatin) or histone methyltransferase inhibitor (DZNep) results in softer nucleus and induces nuclear blebbing. Conversely, treatment with histone demethylase inhibitor (methylstat) increases the rigidity of the nucleus (Stephens et al., 2018). Down-regulation of the Prdm3 and Prdm16 (H3K9 methyltransferases) breaks heterochromatin organization and at the same time leads to atypical nuclear morphology, including defects in nuclear lamina organization and invaginations of the nuclear envelope (Pinheiro et al., 2012). Overexpressing HMGN5 protein destabilizes chromatin through reducing the ability of the linker histone H1 to bind nucleosomes (Rochman et al., 2009). This chromatin decompaction decreases nuclear stability and leads to nuclear blebbing (Furusawa et al., 2015).

The chromatin decompaction in cancer samples are well distinguishable by staining of tissue sections and tumor cells. Differences in the distribution of heterochromatin areas inside the nucleus are clearly visible, which is the basis for the diagnosis of several cancer subtypes (Fischer et al., 2010). The specific feature of cancer cells is formation of aberrant aggregates and decrease or the complete absence of condensed, dark material within cancer nuclei (Gurrion et al., 2017; Xu et al., 2022). The mechanism underlying these changes in heterochromatin organization of cancer cells is not yet fully understood. It can be a result of dysregulation of the tumor-suppressor genes responsible for heterochromatin formation or maintains. For example, BRCA1 functions as a tumor suppressor by maintaining heterochromatin integrity and silencing the expression of non-coding pericentromeric satellite RNAs (Zhu et al., 2011). Unphosphorylated STAT3 possesses noncanonical function of tumor suppression by promoting heterochromatin formation by associating with HP1 (Dutta et al., 2020).

At the same time, lamin gene disruption can be the direct reason of heterochromatin disorganization (Lämmerhirt et al., 2022). Chromatin interact with the nuclear lamina and form lamina-associated domains, containing mostly repressed genes (Steensel and Belmont, 2017). Correct positioning of the lamina-associated domains is important for regulation of gene expression (Ou et al., 2017). The knockout of lamin B1 gene leads to the chromatin decompaction and detachment of lamina-associated domains from the periphery in cultured cells (Chang et al., 2022). There is unclear what exactly happens at lamina-associated domain level during tumorigenesis. However, it is possible to make some assumptions about the behavior of lamina-associated domains in cancer cells from data concerning oncogene-induced senescence of cells. This type of senescence is induced by activation of oncogenes, including BRAF, AKT, E2F1 and cyclin E, and by the inactivation of tumor suppressor genes, including PTEN and NF1 (Liu et al., 2018). Most of the constitutive lamina-associated domains lose contact with the nuclear lamina during oncogene-induced senescence and part of the chromatin from inner nucleus regions moves closer to the nuclear lamina (Lenain et al., 2017).

Taken together, these observations show a complex mutual influence of nuclear lamina and chromatin in the cell nucleus. We believe that future studies will shed light on the root cause of the altered nuclear morphology in cancer cells. It is important as the atypical nuclear morphology can promote cancer cell invasion and metastatic dissemination by facilitate cell squeezing through narrow tissue spaces (Pandya et al., 2017).

### Level 2—Chromosome territories

**FIGURE 1:**
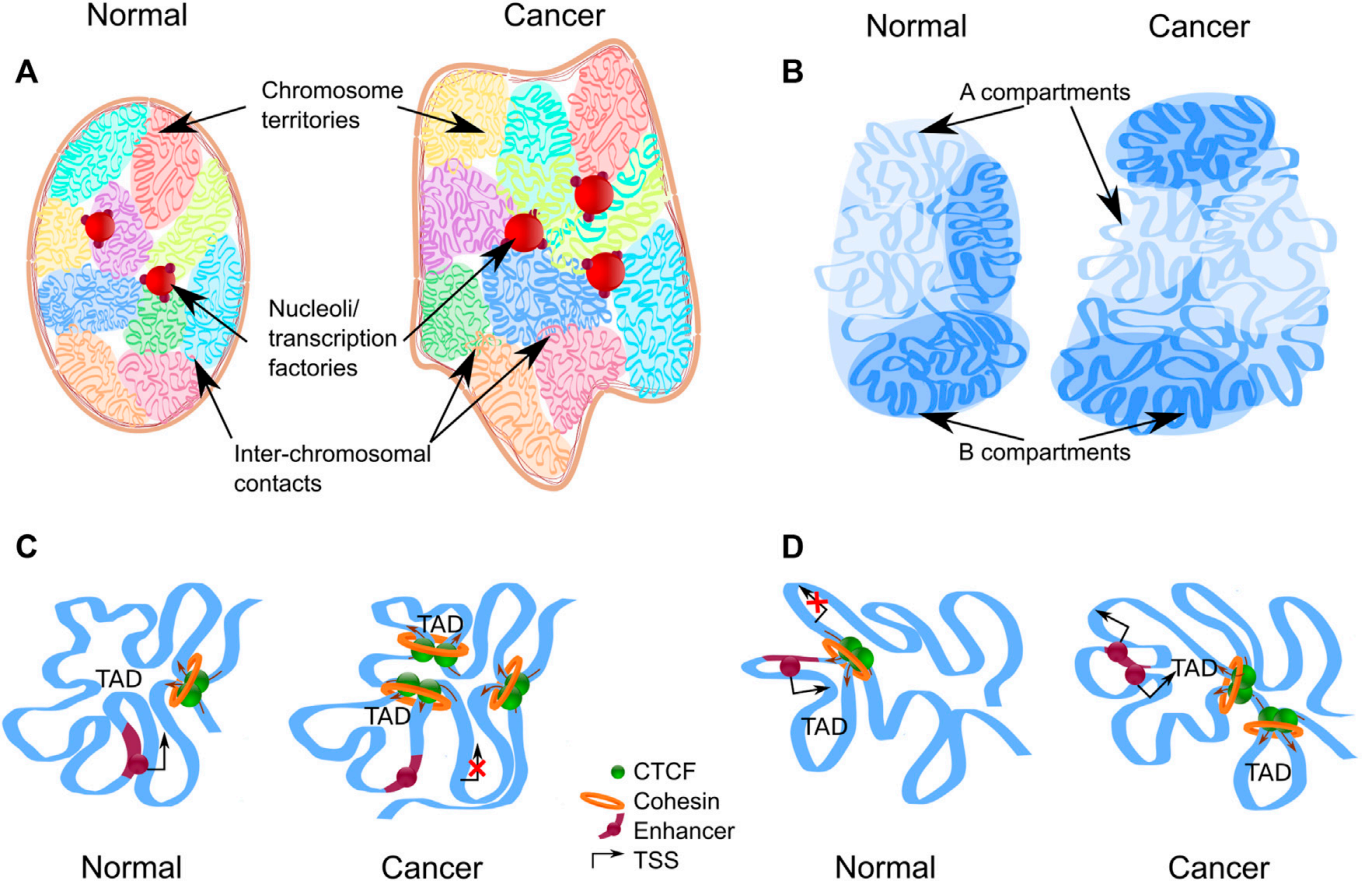
Levels of chromatin architecture organization in normal and cancer cells. **(A)** Chromosome territories change location in the nucleus of a cancer cell. Some chromatin regions can be up-regulated as a result of moving from the silent environment of lamina-associated domains to a nuclear interior. Entanglement between some chromosomes increases in the nucleus of a cancer cell. Chromosome territories repositioning and intermingling can facilitate certain translocation formation. **(B)** Compartment switching is in cancer cells, which is followed by expression alteration. Long-range interactions between different types of compartments are increased in cancer cells. **(C,D)** Topologically associating domains (TADs) reorganize in cancer cells. The appearance of new TAD may destroy the regulation of oncogenes or tumor-suppressor genes **(C)**. Fusion of TADs in different epigenetic states could lead to the formation of new regulatory landscape and upregulation of repressed genes **(D)**.

The interphase nucleus is a highly ordered structure where each chromosome occupies a certain territory without global mixing with its neighbors. In accordance with the changes of nucleus size, the volume of chromosome territory increases in the cancer cell. However, enlargement of chromosome territories and nuclear volume is not proportional; as a result, relative volumes of chromosome territories are smaller in cancer cells than in normal cells (Sathitruangsak et al., 2017). The observed disproportion between the nucleus and chromosome territories volume may be the result of an increase in the DNA-free/poor space in the cancer cell nuclei (Sathitruangsak et al., 2015; Righolt et al., 2016). This space can be occupied by the nucleoli or transcription factories. Аn increase in the number and size of nucleoli is correlated with an increase in tumor aggressiveness (Berus et al., 2020). Chromosomes occupy their territories non-random within interphase nucleus, but according to the gene density; gene-rich chromosome territories are located in the nuclear center, and gene-poor territories are more peripheral (Cremer & Cremer, 2010). Transcriptional activity and replication timing can influence the radial position of chromosome territories (Goetze et al., 2007; Grasser et al., 2008). The specific size and order of chromosome territories are distinct in different cell types (Hepperger et al., 2008) and change during cell differentiation and development (Kuroda et al., 2004; Sehgal et al., 2016; Lomiento et al., 2018). In the same way, chromosome territories change location in the nucleus of a cancer cell, reflecting cancer-specific dysregulation of gene expression (Marella et al., 2009) (Figure 1A). For example, chromosomes 4, 9, 14 and 18 shift to the nuclear center in human myeloma cells compared to lymphocytes; on the contrary, the location of chromosomes 11 and 16 is more peripheral (Sathitruangsak et al., 2017). In breast cancer cells, chromosomes 4, 12, 15, 16 and 21 are significantly more peripherally located compared to normal cells. At the same time, all these chromosomes, except 4, contain a large proportion of downregulated genes in cancer cells (Fritz et al., 2014).

Besides entire chromosome territories changing location within the nucleus simultaneously with the changes in transcription, individual genes also move within chromosome territories and, moreover, loop out. As it was mentioned the gene-poor chromosomes occupy the nucleus periphery; however, the distribution of genes within chromosome territories follows a different logic; namely, active genes are localized more peripherally within the chromosome territories (Torabi et al., 2017). The loop out of active genes from their own chromosome territories leads to mixing of neighboring territories, and together these genes can be organized in transcription factories (Eskiw et al., 2010) or splicing speckles (Brown et al., 2008). There is some evidence that cancer chromatin structure is significantly fluffier, as a result, entanglement between some chromosomes increases (Barutcu et al., 2015; Kim et al., 2022; Yang et al., 2022).

The mechanism of chromosome territories formation is not clear. Condensins I and II play a central role in chromosome condensation during mitosis. In addition, condensin II remains within the nucleus throughout interphase, and contributes to the structure of the chromosomes. Cap-H2 (one of condensin II subunits) is required for chromosome territory formation in Drosophila cells, promotes long distance interactions within the chromosome territory, and, at the same time, prevents chromosomes from intermingling (Bauer et al., 2012; Rosin et al., 2018). In Drosophila cells, Cap-H2 depletion leads to a significant increase in chromosome volume, a decrease in *cis* interactions, and increase in *trans* interactions. As a result of chromosome territories decompaction, translocations frequently occur in Cap-H2 depleted cells (Rosin et al., 2019). In contrast to Drosophila, condensin II functions in the interphase nuclei of mammalian cells are poorly understood. Its knockdown leads to enlarged and misshapen interphase nuclei as a result of impaired chromatin compaction (Fazzio & Panning, 2010). This observation contradicts the recent data showed that MCPH1 protein disturbs condensin II association with chromatin in the interphase by break the SMC2 and NCAPH2 link. Interestingly, loss of MCPH1 induces condensin II dependent premature chromosome condensation (Houlard et al., 2021). Thus, it is possible that condensin II continues chromosome condensation during interphase. The condensation does not reach the mitotic level due to the presence of MCPH1 protein. However, this condensation level is sufficient to maintain chromosome territories. Given all of the above, mutations of the condensin complex genes, which are frequently observed in different cancer types (Leiserson et al., 2015), are likely to be the reason for the changes in chromosome territories in cancer cells. In addition, condensin II mutations compromise genomic safety by affecting telomere stability and chromosome segregation (Wallace et al., 2019; Weyburne & Bosco, 2021). As described above, dysregulation of lamins is often observed in cancer cells and it is possible participant influencing the organization of chromosome territories. Knockout of lamin A/C or B leads to increasing volume of chromosome territories (Camps et al., 2014; Ranade et al., 2017) and chromatin mobility increases as a result of downregulation of lamin B1 (Chang et al., 2022).

Intermingling of chromosome territories observed during malignancy can lead to secondary oncogenic events associated with tumor progression, facilitating translocations between certain chromosomes (Branco & Pombo, 2006), depending on their physical proximity (Roix et al., 2003; Zhang et al., 2012a; Engreitz et al., 2012) (Figure 1A). Redistribution of chromosome territories in myeloma cells causes spatial proximity of chromosomes, which correlates with common myeloma-associated translocations (t(11;14), t(4;14), and t(14;16)) (Sathitruangsak et al., 2017). This correlation of spatial proximity and frequency of chromosomal translocations can also be demonstrated at the scale of individual genes. For example, the frequency of fusion between FGFR3 (chromosome 4) and IGH (chromosome 14) genes is four times higher than fusion of MAF (chromosome 16) and IGH (chromosome 14) in the myeloma patient population. In accordance with it, FGFR3 loops out from its chromosome territory significantly more frequently than MAF, which occupies predominantly internal locations in healthy B-cells. As a result, FGFR3 co-locates with IGH significantly more frequently. Another well-studied example highlighting the mechanistic role of spatial proximity in carcinogenic chromosomal translocations is MYC-IGH fusion in Burkitt lymphoma. It was shown that Tat protein from human immunodeficiency virus induces a prolonged MYC relocalization next to IGH in circulating B-cells, significantly increasing the risk of translocation between these genes. This observation explains elevated levels of incidence of Burkitt lymphoma in human immunodeficiency virus infected individuals (Germini et al., 2017). Finally, analysis of thousands of induced translocations in human pro-B cells shows that selection of translocation partners is guided by the spatial proximity of the loci (Zhang et al., 2012b). It is apparent that physical proximity is not the only factor influencing certain translocation formation; the others include the numbers of double-strand breaks, the probability of their arising at a particular site, and their mobility.

To sum up, changes in chromosome territories position and volume, as well as relocation of specific loci outside their chromosome territories and increasing intermingling are observed in cancer cells (Table 1). These changes may result in alteration of gene expression or promote specific chromosomal translocations essential for cancer progression.

**TABLE 1:** Chromatin architecture alterations in cancer.

Levels of chromatin architecture	Features	Directions of changes in cancer cells
1. Nucleus	Size	Increase
	Morphological disorder	Increase
2. Chromosome Territories	Size	Increase
	Displacement	Increase
	Isolation	Decrease
3. Compartments	Conservatism	Decrease
	Displacement	Increase
	Isolation	Decrease
4. Topologically associating domains	Size	Decrease
	Number	Increase
	Isolation	Decrease

### Level 3—Compartments

Genomic compartments can be distinguished within chromosome territories as clusters of specific chromatin subtypes. At low resolution, compartments can be identified as megabase-scale genomic regions that are either euchromatic with high gene density (A-type) or heterochromatic with low gene density (B-type). The chromatin interactions between the different types of compartments are dramatically reduced. Increase in resolution provides a more detailed view of chromatin composition of compartments, allowing to identify spatially segregated subtypes of active- and repressed chromatin (Rao et al., 2014).

It is assumed that chromatin compartments allow to locally increase the concentration of enzymes required for specific molecular processes, such as transcription initiation and elongation, RNA splicing, gene silencing, etc. (Mora et al., 2022). Compartments are established and maintained due to weak multivalent interactions of chromatin proteins, a process known as liquid-liquid phase separation (Kantidze & Razin, 2020). Distribution of compartments is affected by changes in expression levels of phase-separating proteins, patterns of their binding to DNA, post-translational modifications or mutations. Distribution of compartments changes during of cellular differentiation and development. For example, more than a third of A/B compartments switch from one type to another during human embryonic stem cells differentiation (Dixon et al., 2015). Similarly, compartment switching is observed in the cancer cells (Barutcu et al., 2015; Vilarrasa-Blasi et al., 2021) (Figure 1B): about 20% of loci switch compartment from A to B and B to A, (Adeel et al., 2021; Kim et al., 2022; Yang et al., 2022). The relatively low proportion of the genome undergoing this switching can be explained by the fact that chromatin in the A and B compartments can be additionally divided according to its plasticity degree (Liu et al., 2021): there are some areas that change their state faster and more willingly than others. About 19% of chromatin attributed to A compartment can be considered as intermediate compartment “I” that can interact with both canonical compartments in normal cells and predominantly with B in cancer (Johnstone et al., 2020).

One mechanism explaining compartment switching in cancer cells is overexpression of an oncogenic transcription factor, which is normally absent or expressed at low level. The protein binds its DNA targets, and engages them to an active compartment. This results in a dramatic shift of DNA compartmentalization profile, and accompanied changes of gene expression and cellular properties. For example, activation of the KLF5 transcription factor results in spatial clustering of its binding sites, recruitment of CBP/EP300, BRD4 and eventually Polymerase II complexes into these clusters, leading to numerous transcriptional changes (Liu et al., 2020). This oncogenic activation of KLF5 was reported in multiple epithelial cancer types, highlighting the role of compartment switching in cancer. Similarly, mutations activating Notch result in repositioning of Notch-bound enhancers, leading to transcriptional activation of its targets in triple-negative breast cancer and B-cell lymphoma (Petrovic et al., 2019).

Another mechanism altering chromatin clustering and causing transcription changes in cancer cells is associated with gain- or loss-of-function mutations of architectural proteins, which affect their ability to form chromatin condensates (Ahn et al., 2021; Jevtic et al., 2022). It is assumed that multivalent interactions of the unstructured domains of peptides, called intrinsically disordered domains (IDRs), allow formation of liquid droplets within nuclear space. Concentration of IDR-containing peptides, their interaction partners and DNA loci associated with these proteins substantially increases within these droplets. There are several examples showing that changes of these domains affect nuclear distribution, sequence specificity, and functional activity of proteins, even if their catalytic and DNA-binding domains remain unmodified. For example, a recent study showed that fusion of the human nucleoporin IDR and HOXA9 transcription factor promote the formation of nuclear condensates. The chimeric HOXA9 transcription factor has a unique DNA-binding profile that strongly differs from wild-type protein and, therefore, its expression results in transcriptional changes and oncogenic transformation in haematopoietic stem and progenitor cells (Ahn et al., 2021). Importantly, the authors showed that treatment of cells with the 1,6-hexanediol, a chemical agent that disrupts hydrophobic interaction-induced phase separation assemblies, suppressed the ability of the fused protein to bind DNA and activate transcription. Thus, the authors showed that IDR-mediated phase separation plays an essential role in oncogenic transformation. Moreover, fusions of nucleoporin IDR and different transcription activator domains were detected in several human hematological malignancies (Jankovic et al., 2008; Wang et al., 2009; Gough et al., 2011; Mendes & Fahrenkrog, 2019), suggesting a common mechanism underlying the formation of these tumors.

Observed compartment switching may influence gene expression in cancer cells. The expression of many genes changes concordant with compartments switching, genes are most likely down-regulated after switching from A to B compartment and *vice versa* up-regulated after B to A switching. At the same time, there are some examples showing that getting into the active compartment is not always accompanied by the activation of gene expression (Nagai et al., 2019; Guo et al., 2021). It can be explained by complexity of transcription regulation network and involvement of other players in the regulation of such genes. Gene ontology analysis shows enrichment in genes of cellular components and different molecular pathways, including cancer-related genes, among the differentially expressed genes located in switched compartments (Adeel et al., 2021; Ren et al., 2021; Kim et al., 2022). For example, B to A compartment switch contributes to up-regulation of the HOX genes in glioblastoma (Yang et al., 2022). On the contrary, the transition of compartment A containing Forkhead box O transcription factor (FOXO1) gene, which have tumor-suppressor functions (Fu and Tindall, 2008a), to compartment B results in loss of H3K27ac upstream of the gene and downregulation of its expression (Liu et al., 2021). However, it is often impossible to distinguish if changes in gene expression occur as a result of compartment switching or initiate it.

The compartment switching alters genomic distribution of somatic mutation. The somatic mutation rate is higher in heterochromatic regions of cancer genomes and the reason remains obscure. Differences in the DNA repair complex availability or an increased effect of mutagens on the nuclear periphery can make a contribution (Schuster-Bockler and Lehner, 2012). The mutational load changes dramatically near boundaries between transcriptionally distinct domains in cancer cells. Moreover, after compartment switching the somatic mutation rate depends on the new compartment status only (Fortin & Hansen, 2015; Akdemir et al., 2020a). It is supposed that compartment switching can occur relatively early in the development of cancer, for example as a result of mutations in the chromatin remodeling enzyme genes (Chen et al., 2016). Subsequently, this switching can lead to a change in the rate of mutation in the affected areas (Fortin & Hansen, 2015).

In addition to switching, compartments undergo a global relocalization in tumor cells. Because A compartments are considered as active gene-rich chromatin, they occupy the nuclear inner regions in normal cells; on the contrary, B compartments are located in the nuclear periphery (Stevens et al., 2017; Falk et al., 2019). However, large heterochromatin foci were found in the nucleus interior in cancer cells (Johnstone et al., 2020). Second, intensification of intermingling observed at the level of chromosome territories, can also be revealed at the compartment level in cancer cells. Long-range interactions between compartments A and B are increased (Johnstone et al., 2020; Martin et al., 2022).

Since histone modifications play an important role in the formation of A/B-compartments, it is not surprising that mutations of chromatin modifying enzymes results in redistribution of compartments. For example, SETDB1 is a histone H3K9 methyltransferase, which is often up-regulated in different types of tumors (Lazaro-Camp et al., 2021). In lung cancer cells, the inactivation of the SETDB1 gene leads to recovery features of a normal epithelial phenotype, including an increase in spatial cis-contacts within the B-compartment and a decrease in inter-compartment interactions (Zakharova et al., 2022).

Compartment switching and relocation, intensification of atypical long-range interactions between different types of compartment are observed in cancer genome (Table 1). In addition to its effects on transcription, reorganization of compartments may influence rates of chromosomal translocations, since 3D genome organization and spatial proximity influence on the probability of chromosomal rearrangements and translocations genome-wide (Zhang et al., 2012a). Thus, the A/B compartments switching and intermingling as well as chromosome territories relocation can facilitate certain translocations in cancer cells.

### Level 4—Topologically associating domains

Going to the next level of genome organization, topologically associating domains (TADs) can be distinguished as the regions of chromatin with a high grade of self-interaction. As well as other genes, proto-oncogenes and tumor-suppressors are sequestered in such domains, which borders protect these genes from miscellaneous enhancer interactions (Hnisz et al., 2016). Thus, TAD boundaries disturbance can lead to gene dysregulation and cancer development (Wang et al., 2021; Zhang et al., 2022). However, even if TAD boundary disturbance is not a driver mutation, the domain level of chromatin organization is undergoing global changes in cancer cells, and about 30% TADs are reorganized. In the altered regions, there are more TADs with smaller sizes (Taberlay et al., 2016; Nagai et al., 2019) (Figure 1C). The fact that the total majority of the boundaries presented in the normal cells are retained after malignancy (Taberlay et al., 2016; Du et al., 2021) may indicate that there is a partitioning of the existing TADs, rather than the arising of new ones. A clear confirmation of this statement is the isolation of 520 large TADs in normal prostate cells, which corresponds to 850 smaller TADs in cancer cells (Rhie et al., 2019). The changes in the domain boundaries concentrate within the loci switching compartments, implying that the local boundary changes are related to the global chromatin rearrangement.

TAD boundaries can change not only their position, disappearing or appearing in new loci, but also change insulator properties in cancer cells (Johnstone et al., 2020; Kim et al., 2022). Unlike the compartment level of the chromatin organization, there is no uniform trend towards a weakening of insulation score and an increase in contacts between neighboring TAD; rather, a total interaction change occurs (Guo et al., 2021; Kim et al., 2022). Thus, newly TAD boundaries appearing in cancer cells may be clearly manifested but not stably maintained. The insulation score of TAD boundaries increases more frequently for domains switched from B to A compartments, and *vice versa*, the score decreases when TAD moves from A to B (Guo et al., 2021). Altogether, it suggests synergistic chromatin structure alteration at the TAD and compartment levels.

TAD changes arise in the cancer genome through a variety of mechanisms including somatic mutation, CTCF binding site alterations, CTCF inferiority. The genome distribution of point mutations is closely related to the spatial chromatin organization with increasing probability near the TAD boundaries and especially around the boundaries between transcriptionally distinct domains (Wu et al., 2017; Akdemir et al., 2020b). TAD boundaries of cancer cells are also affected by different types of somatic structural variations. Duplications are more frequent than deletions. In contrast, deletions more likely occur within the same domain, and do not affect its boundary. (Akdemir et al., 2020a). At the same time, there is relation between the strength of the TAD boundary and the probability of a structural variant modifying this boundary occurrence. The deletion frequency decreases with increasing boundary strength, whereas duplications affected strong boundaries are more frequent. As strong TAD boundaries are associated with super-enhancers, these cis-regulatory elements are often affected by duplications in cancer cells (Gong et al., 2018), leading to dysregulation of oncogenes. For example, сo-duplication of well-known oncogene MYC and its super-enhancers located near strong TAD boundary is often found in different cancer types and accompanied by MYC overexpression (Zhang et al., 2016; Gong et al., 2018; Xing et al., 2019).

Fusion of TADs in different epigenetic states could lead to the formation of new regulatory landscape and upregulation of repressed genes (Figure 1D). Whereas several computational models were recently proposed to infer chromatin architecture of the rearranged loci (Belokopytova and Fishman, 2020), predicting gene expression for these new regulatory landscapes is challenging. For example, WNT4 expression was 37-fold increased in a malignant lymphoma with deletion of nearby TAD boundary compared with lymphomas without such deletion. It is pertinent to note, only 25% cases of boundary deletion between active and repressed domains lead to more than two-fold expression increases (Akdemir et al., 2020b). In general, although a chromosomal rearrangement affecting the TAD boundaries can induce expression changes (Figures 1C,D), at the moment it is not possible to identify a clear concordance between the rearrangement and direction and strength of the expression changing. This suggests the complexity of gene regulation in cancer genomes.

Another mechanism of TAD disturbance observed in cancer cells is CTCF binding change. CTCF is one of the key players establishing chromatin organization. It is a DNA-binding protein required to maintain genome architecture by mediating both short and long chromosome contacts (Phillips & Corces, 2009; Ghirlando & Felsenfeld, 2016). The reduced loop formation as well as TAD structure changes can be the result of CTCF occupancy distortion. Indeed, chromatin of some cancer types is characterized by total TAD weakening. In this case, CTCF leaves the boundaries of the domains, while its expression does not change. At the same time, the chromatin of these boundaries has reduced accessibility. The converse statement is also true for other tumors; CTCF increases the occupancy at the boundaries of more distinct domains, that is closely coupled with the gain of chromatin accessibility (Kim et al., 2022). The wide-scale comparison of lost/gained CTCF binding shows that the lost binding sites are common for various cancer types, whereas gained sites show cancer-type specificity (Fang et al., 2020).

The CTCF occupancy reduction may happen due to mutations in the binding sites, which undergo a high mutational load in different types of cancer (Katainen et al., 2015; Guo et al., 2018). The most described mutations can reduce binding of CTCF (Kaiser et al., 2016), it supposes there is selection pressure on such variants during tumorigenesis. At the same time, it is not possible to establish a direct connection between most of these mutations and cancer development. However, there are some examples where disruption of an insulator sites have a functional role and act as drivers. Recurrent deletions in T-cell acute lymphoblastic leukemia affect CTCF binding sites and cause changes in the expression of LMO2 and TALI oncogenes (Hnisz et al., 2016). Another example is mutation of the insulator near the TGFB1 gene in melanoma. As a result, TAD boundary disruption occurs, which leads to TGFB1 up-regulation and increasing of cell proliferation by the activation of the transforming growth factor-β (TGF-β) signaling pathway (Liu et al., 2020). It is known that mutations of CTCF sites in the melanoma cells occur due to uneven nucleotide excision repair across the motif (Sabarinathan et al., 2016). This erroneous repair is caused by low DNA accessibility precisely at the sites occupied by CTCF (Poulos et al., 2016).

High levels of DNA methylation are associated with nucleosome compaction and reduced chromatin accessibility for different TFs, including CTCF (Hark et al., 2000; Choy et al., 2010; Fang et al., 2020). The genome-wide comparison between CTCF occupancy and DNA methylation indicates that in cancer cells, CTCF losses are accompanied by the DNA methylation increase, whereas a lot of gained CTCF are in the demethylated region (Fang et al., 2020). It supposes that at least a portion of the cancer-specific changes in CTCF occupancy is related to a change in DNA methylation of the binding sites. Genomes of some cancers are globally hypermethylated as a result of demethylases inhibition. In the case of isocitrate dehydrogenase (IDH) mutant gliomas the affected cells accumulate 2-hydroxyglutarate, which disrupts the function of TET family of 5′-methylcytosine hydroxylases, which takes part in the removal of DNA methylation (Kohli and Zhang, 2013). Besides hypermethylated CpG islands, IDH mutations lead to hypermethylation of CTCF binding sites. CTCF can not occupy the methylated binding sites and there is disturbance of boundary elements separating TADs genome-wide. As a result, in IDH-mutant glioma the disturbance of PDGFRA-domain boundary induces aberrant contacts between PDGFRA gene and FIP1L1 enhancer, which causes the activation of this oncogene (Flavahan et al., 2016). The similar mechanism underlying formation SDH-deficient tumors, where about 5% of CTCF-sites are lost due to the increasing DNA methylation. As result, the insulation in the FGF and KIT TADs is disturbed and the super-enhancers interact with their promoters and powerfully activate expression (Flavahan et al., 2019).

Thus, changes in TAD boundaries potentially underlie tumorigenesis by altering the spatial architecture of regulatory elements and proximity to genes, leading to aberrant expression of cancer related genes.

## Conclusion

During the last decades, key principles of chromatin organization and their role in genome function have been gradually discovered. At the moment, there is an understanding of the importance of chromatin folding in the processes of development and diseases. It makes possible to integrate knowledge about the principles of chromatin architecture organization into a comprehensive understanding of cancer development and the risks of its progression. Results discussed here suggest that malignization affects every level of genomic organization and chromatin organization alterations can contribute to cancer progression. The main changing vector that can be found at different levels is chromatin mixing (Table 1). This is reflected in heterochromatin decompaction and redistribution, which is proved by microscopic observations and Hi-C data. The last one allow to find out not only compartment A and B redistribution, but also increasing long-range interactions between different type of compartments. The increased intermingling of chromosome territories and its relocation in cancer cells continue the indicated trend. Although, it still is unclear whether the reorganization of spatial chromatin architecture is a result of a global change in the expression of many genes in tumor cells or is an instigator of such changes. It is highly likely that understanding how malignancy affects chromatin architecture will be important for getting a more complete picture of cancer development and progression and its respond to therapy.
